# AI‐driven simplification of surgical reports in gynecologic oncology: A potential tool for patient education

**DOI:** 10.1111/aogs.15123

**Published:** 2025-05-14

**Authors:** Maximilian Riedel, Bastian Meyer, Raphael Kfuri Rubens, Caroline Riedel, Niklas Amann, Marion Kiechle, Fabian Riedel

**Affiliations:** ^1^ Department of Gynecology and Obstetrics, TUM University Hospital Technical University Munich (TU) Munich Germany; ^2^ Institute of Computational Biology Helmholtz Zentrum München, German Research Center for Environmental Health Neuherberg Germany; ^3^ Department of Medicine III, Hematology and Oncology TUM University Hospital, Technical University Munich Munich Germany; ^4^ TUM School of Medicine Technical University of Munich Munich Germany; ^5^ Department of General Internal Medicine and Psychosomatics Heidelberg University Hospital Heidelberg Germany; ^6^ Department of Gynecology and Obstetrics Friedrich–Alexander‐University Erlangen–Nuremberg (FAU) Erlangen Germany; ^7^ Department of Gynecology and Obstetrics Heidelberg University Hospital Heidelberg Germany

**Keywords:** AI, GPT‐4, gynecology, large language models, patient education, surgery

## Abstract

**Introduction:**

The emergence of large language models heralds a new chapter in natural language processing, with immense potential for improving medical care and especially medical oncology. One recent and publicly available example is Generative Pretraining Transformer 4 (GPT‐4). Our objective was to evaluate its ability to rephrase original surgical reports into simplified versions that are more comprehensible to patients. Specifically, we aimed to investigate and discuss the potential, limitations, and associated risks of using these simplified reports for patient education and information in gynecologic oncology.

**Material and Methods:**

We tasked GPT‐4 with generating simplified versions from *n* = 20 original gynecologic surgical reports. Patients were provided with both their original report and the corresponding simplified version generated by GPT‐4. Alongside these reports, patients received questionnaires designed to facilitate a comparative assessment between the original and simplified surgical reports. Furthermore, clinical experts evaluated the artificial intelligence (AI)‐generated reports with regard to their accuracy and clinical quality.

**Results:**

The simplified surgical reports generated by GPT‐4 significantly improved our patients' understanding, particularly with regard to the surgical procedure, its outcome, and potential risks. However, despite the reports being more accessible and relevant, clinical experts highlighted concerns about their lack of medical precision.

**Conclusions:**

Advanced language models like GPT‐4 can transform unedited surgical reports to improve clarity about the procedure and its outcomes. It offers considerable promise for enhancing patient education. However, concerns about medical precision underscore the need for rigorous oversight to safely integrate AI into patient education. Over the medium term, AI‐generated, simplified versions of these reports—and other medical records—could be effortlessly integrated into standard automated postoperative care and digital discharge systems.

## Graphical abstract

Artificial intelligence (AI)‐driven simplification of medical reports holds promise for improving patient education. However, careful attention must be paid to ensuring accuracy and completeness. Use of AI technologies in healthcare will continue to expand but will require rigorous oversight and ongoing research to fully realize their potential in clinical practice.

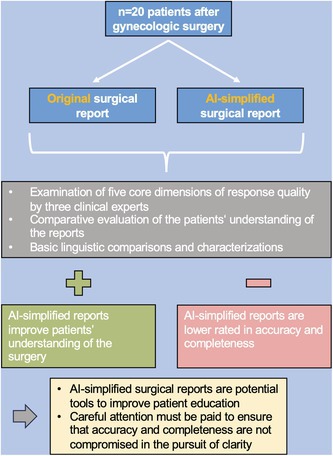

**Abbreviations**
AI: artificial intelligence
GPT: Generative Pretraining Transformer
LLM: large language model

**Key message**

Simplified surgical reports generated by Generative Pretraining Transformer‐4 improve patient understanding of procedures and outcomes in gynecologic oncology. However, concerns about medical precision underscore the need for rigorous oversight to safely integrate artificial intelligence into patient education.

## 1 INTRODUCTION

Generative Pretraining Transformer 4 (GPT‐4) is a recent version of a modern and complex large language model (LLM) developed and first released by OpenAI in 2022. Trained on extensive collections of text data, GPT‐4 has been optimized to simulate natural, human‐like conversations. It uses deep learning algorithms to analyze language patterns, syntax, and the semantics of natural language.^1^, ^2^ The model selects the most likely next token from a large pool of sub‐word token embeddings, effectively mimicking human language patterns and providing relevant information or assistance. The user‐friendly design of its browser‐based online application “ChatGPT” enables effortless interaction and conversation and requires minimal to no training or technical expertise.^3^ In addition to applications in customer service, content generation, and translation,^4^ LLMs show significant potential for use in medicine.^5^

Exploratory studies have evaluated the ability of GPT to address medical questions and problems. It demonstrated promising results on original exam questions from the United States Medical Licensing Examination (USMLE), where it even partially outperformed the average scores of medical students.^6^, ^7^ We observed similar positive outcomes in our own analysis of the obstetrics and gynecology course exams at our institution and original German medical state exams questions.^8^ Additionally, we investigated GPT‐4's ability to assist in therapeutic decision‐making by using data from original gynecologic and breast cancer interdisciplinary tumor boards. Our findings demonstrated a considerable alignment between GPT‐4's recommendations and the actual consensus reached by the tumor boards (unpublished data, currently under review).

The use of LLMs and artificial intelligence (AI) in the medical field is expected to grow significantly in the coming years.^2^, ^5^, ^9^ It is likely that the number of patients who rely on these technologies to find answers for medical questions or to better understand their personal health data or records will grow. This is especially relevant in the very sensitive field of gynecologic oncology.^10^ To the best of our knowledge, this is the first study to explore GPT‐4's ability to rephrase anonymized surgical reports into versions that are more accessible and easily understandable for non‐professionals. This is particularly important, as clear and precise communication is essential to the doctor–patient relationship and plays a pivotal role in the quality of healthcare delivery.

The study focused on addressing the following three core questions:

1. How effectively can GPT‐4 simplify detailed medical content for patients while preserving critical information and avoiding the omission of essential details?
2. What are the potential benefits and limitations of GPT‐4 to rephrase complex medical information for patient education and information?
3. What potential risks are associated with the use of GPT‐4 in this context, specifically concerning the accuracy and clarity of patient communication?

## 2 MATERIAL AND METHODS

### 2.1 Data acquisition and processing

A total of *n* = 20 gynecologic patients were randomly selected between January and August 2024 from the Department of Obstetrics and Gynecology at the University Hospital of the Technical University of Munich, Germany, after undergoing open abdominal surgery for expected or assumed gynecological tumors or malignancies. See Table 1 for the summarized list of the surgical procedures. We excluded patients who suffered from a complicated postoperative course preventing the completion of the questionnaire.

**TABLE 1:** List of the surgical procedures of each participating patient.

Number	Operation
1	Laparotomy for GIST
2	Laparotomy for fibroids of the uterus
3	Laparotomy for carcinosarcoma of the uterus
4	Laparotomy for fibroids of the uterus
5	Laparotomy for ovarian cancer
6	Laparotomy for borderline ovarian tumor
7	Laparotomy for ovarian cancer
8	Laparotomy for ovarian cancer
9	Laparotomy for large ovarian cyst
10	Laparotomy for fibroids of the uterus
11	Laparotomy for fibroids of the uterus
12	Laparotomy for ovarian cancer
13	Laparotomy for ovarian cancer
14	Laparotomy for endometrial cancer
15	Laparotomy for ovarian cancer
16	Laparotomy for ovarian cancer
17	Laparotomy for fibroids of the uterus
18	Laparotomy for endometrial cancer
19	Laparotomy for large ovarian cyst
20	Laparotomy for fibroids of the uterus

For each patient, the anonymized surgical report was submitted to GPT‐4 in its original German version. No changes were made to the original report, leaving abbreviations, spelling errors, and ambiguities in the prompt to reflect a real‐world setting. GPT‐4 was subsequently tasked to generate a “simplified version of the report to improve understanding for non‐professionals.” We standardized our prompt in line with Gilson et al.^7^ This standardized data preprocessing was essential, given the substantial impact that the phrasing of prompts can have on the LLM's generated output.^11^, ^12^, ^13^ An exemplary AI‐modified surgical report translated in English can be found in the Appendix S1.

### 2.2 Data analysis

We implemented a two‐step method for data analysis. First, all original and simplified surgical reports generated by GPT‐4 were presented to three clinical experts specializing in gynecology or gynecologic oncology. These physicians were not involved in the primary surgery. We examined GPT‐4's responses using five core dimensions of response quality, originally proposed by Richard Wang and Diane Strong.^14^ These dimensions are commonly utilized to evaluate the dependability and accuracy of data acquisition. By adhering to these established categories, our evaluation of the output by GPT‐4 was consistent with accepted data quality benchmarks. We used this evaluation also in our previous research to examine GPT‐4's performance in answering medical exam questions^8^ and its potential role in supporting clinical decision‐making in multidisciplinary tumor board discussions (unpublished data).

We differentiated the following five dimensions of data acquisition:

1. *Ease of understanding*: Was the answer clearly and precisely formulated in a way that was easy to understand?
2. *Concise representation*: Was the answer clearly structured and divided into sections that facilitated readability?
3. *Accuracy*: Did the facts mentioned in the answer correspond to the current scientific literature? Were the statements logical and understandable?
4. *Completeness*: Was the answer complete, and were all aspects of the question adequately addressed? Was important information omitted, or were there unnecessary details?
5. *Relevance*: Was the answer directly related to the question asked, or was there any ambiguity in the answer?

For this analysis, three medical experts independently evaluated GPT‐4's responses using the five aforementioned criteria in a five‐point Likert scale. The scale ranged from 1 = “*completely disagree*” to 5 = “*completely agree*.”

In the second phase of the assessment, we provided each patient both her original surgical report and the simplified version generated by GPT‐4. The patients were not informed which report was original and which was AI‐generated. The order in which the reports (original and GPT‐generated) were presented in the questionnaire was determined at random. We handed out three separate questionnaires along with the surgical reports. The first questionnaire included biographical questions (*n* = 3), questions regarding the surgical procedure (*n* = 8) and the patient's prior knowledge of it, as well as questions on the patients' general internet usage and prior experience with AI or LLMs (*n* = 6). The questions were a mix of dichotomous or classification questions with a varying number of answer options (the translated questionnaire can be found in the Appendix S2). Patients completed the other two questionnaires immediately after reviewing each surgical report. Both questionnaires featured the same eight questions and five‐point Likert scales to assess the patients' understanding of the report and its effectiveness in clarifying the surgical procedure, associated risks, and the outcome.

To further understand how GPT‐4 responded to our prompts, we selected a representative patient case involving a laparotomy for suspected cancer. We analyzed manually both the original and simplified versions, focusing on basic linguistic characteristics, including word count, average sentence length, the number of technical terms, abbreviations or acronyms, as well as the use of main clauses or subordinate clauses.

### 2.3 Statistical analyses

The data were evaluated using Excel (Version 16.78, 2023, Microsoft) or Prism (Version 10.3.1, 2024, GraphPad). Unpaired *t*‐tests were used to compare the patients' and clinical experts' responses to the original and GPT‐4‐generated surgical reports via five‐point Likert scale ratings. The ratings for the original and GPT‐4‐generated surgical reports were collected from independent samples, and the variables were interval scaled. The aggregation of responses and our sufficiently large sample size allowed us to treat the data as approximately normally distributed. Prior to conducting the *t*‐test, we confirmed the assumption of normal distribution graphically by using the Q‐Q plot and verified the homogeneity of variances. All *p*‐values <0.05 were defined as statistically significant. Tables were generated using Word (Version Office 2405, 2024, Microsoft). Figures were generated in Prism (Version 10.3.1, 2024, GraphPad).

## 3 RESULTS

### 3.1 Demographics and general knowledge about the operation

The *n* = 20 participating patients had a mean age of 61 years (Standard deviation (SD) = 10 years). With regard to their highest level of education, 60% (*n* = 12) reported secondary school, 5% (*n* = 1) held a high school diploma, and 35% (*n* = 7) a university degree. Participation in the study occurred on average 3.5 days (SD = 2.7 days) postsurgery. All patients (*n* = 20) stated that they felt “*well*” or “*very well*” informed about the operation prior to its beginning. Similarly, 95% (*n* = 19) reported feeling “*very well*” or “*well*” informed about the course and outcome of the surgery afterwards. The majority (80%; *n* = 16) did not intend to read the original surgical report to gain a better understanding of the operation and its outcome.

### 3.2 Usage of the internet and experiences with AI/LLMs

Most participants (80%; *n* = 16) reported regular internet use at least several times a week. The most common purposes for internet use were emails (95%; *n* = 19), online news (80%; *n* = 16), health‐related research (30%; *n* = 6), online shopping (20%; *n* = 4), or social media (20%; *n* = 4). Although 40% (*n* = 8) reported having heard of ChatGPT or GPT‐4 before the study, 85% (*n* = 17) believed that the role of AI in medical care and treatment will increase in the future.

### 3.3 Assessment of the surgical reports by clinical experts

**FIGURE 1:**
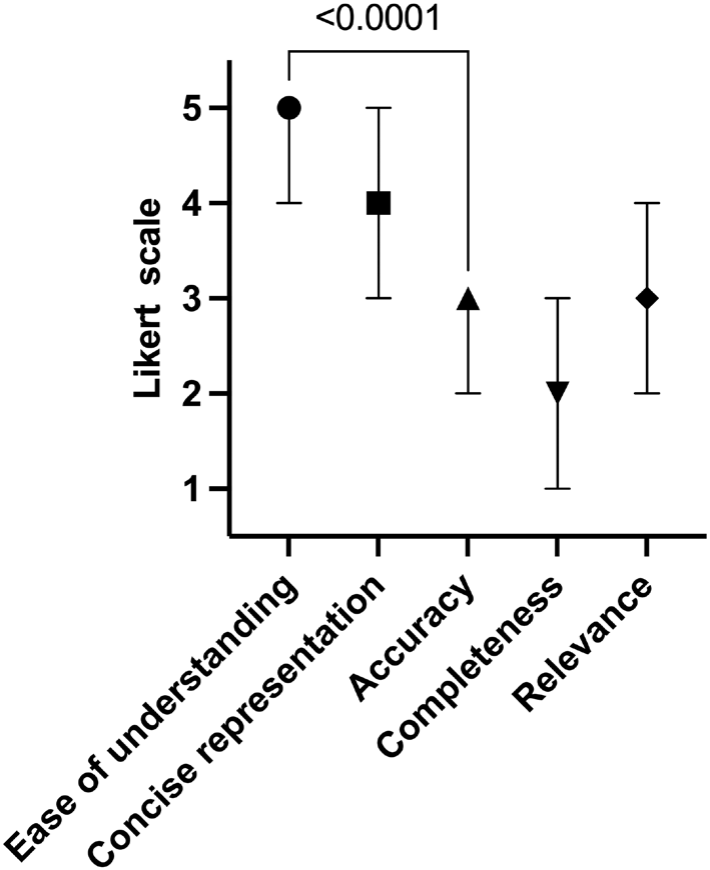
Chart illustrating the qualitative evaluation of GPT‐4's generated surgical reports from *n* = 20 patients based on the established five data quality categories. The median values and range, as evaluated by three clinical experts, are presented in a five‐point Likert scale ranging from 1 = “*fully disagree*” to 5 = “*fully agree*.” Statistical analysis was performed using an unpaired *t*‐test.

In terms of the core data quality dimensions described above, GPT‐4 excelled in *ease of understanding* (median Likert scale of 5.0; range = 1.0) and *concise representation* (median Likert scale of 4; range = 2.0). It also received high scores for *relevance* (median Likert scale of 3.0; range = 2.0) indicating how well the answer aligns with the question asked. However, the performance of the model was rated lower in terms of *accuracy* (median Likert scale of 3.0; range = 1) and *completeness* (median Likert scale of 2; range = 2), which focus on clinical precision and depth of medical information (Figure 1).

### 3.4 Patients' experiences with the simplified surgical report

**FIGURE 2:**
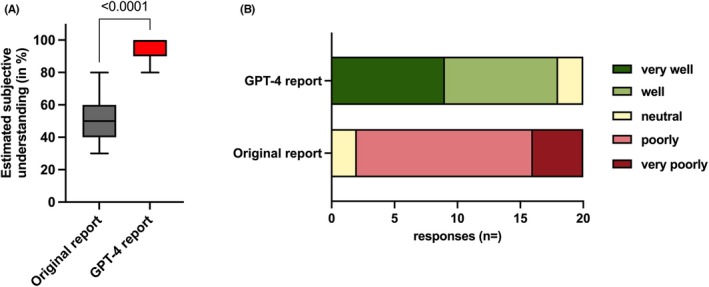
(A) Box plot depicting the responses from *n* = 20 patients how they estimated their subjective understanding (from 0% to 100%) of the original and GPT‐4‐generated surgical reports. Statistical analysis was performed using an unpaired *t*‐test. (B) Chart illustrating the responses from *n* = 20 patients to the question: “How well or poorly did you understand the surgical report?” with varying color gradations on a five‐point Likert scale from “*very poorly*” to “*very well*.”

The majority of our patients indicated a significantly (*p* < 0.0001) better understanding of the simplified version compared with the original report (Figure 2A); 90% (*n* = 18) noted that they have understood the GPT‐4‐generated surgical report “*well*” or “*very well*,” whereas 90% reported a “*poor*” or “*very poor*” understanding of the original reports (Figure 2B).

**FIGURE 3:**
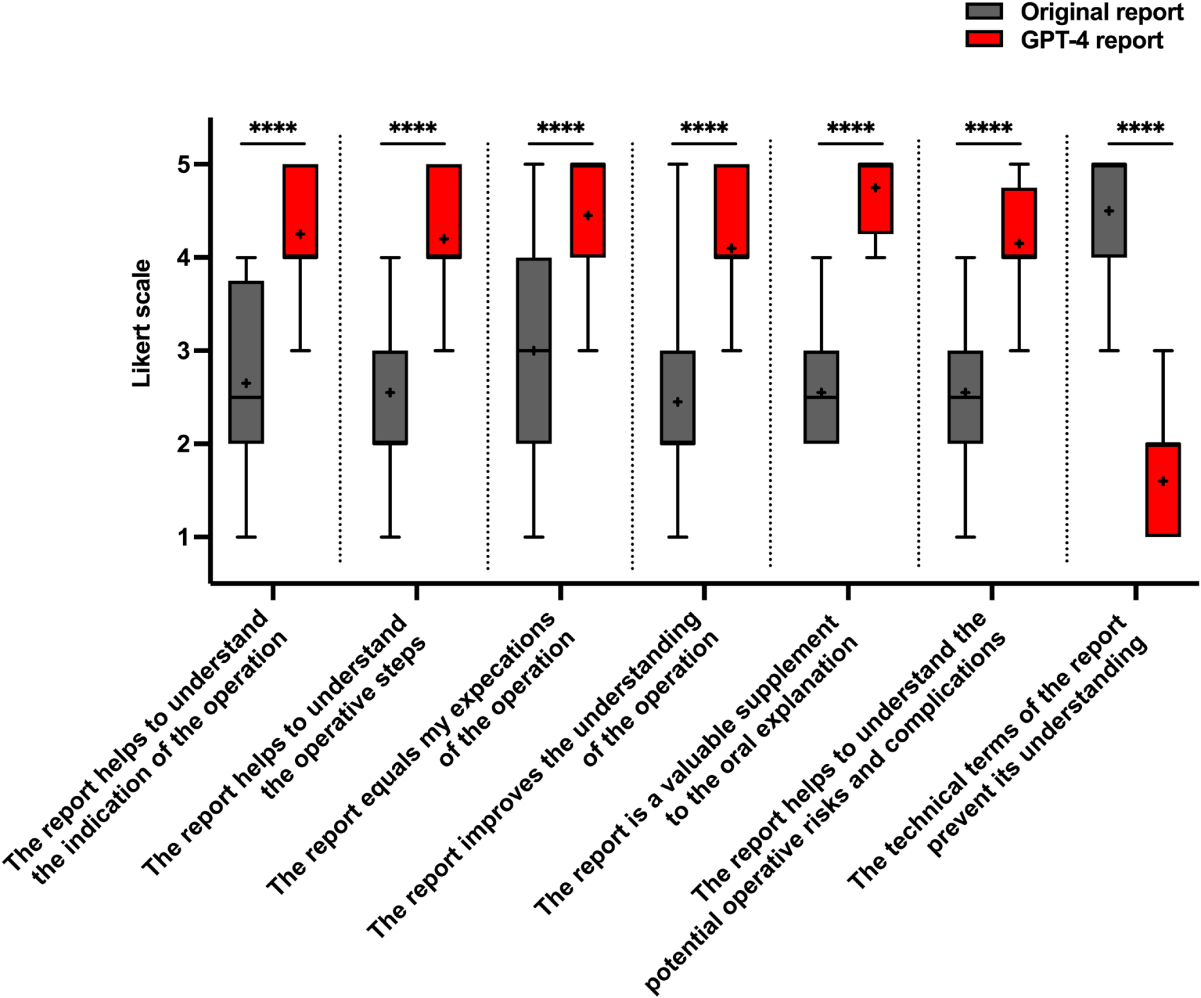
Box plot with “whiskers” illustrating the mean five‐point Likert scale ratings from “*fully disagree*” to “*fully agree*” with the respective statements for both the original (gray) and GPT‐4‐generated (red) surgical reports. The central box spans from the 25th to the 75th percentile, with a horizontal line inside the box marking the median value. “Whiskers” extend from the box to the min. and max. data points. A “+” marks the mean value for each item. Statistical analysis was performed using an unpaired *t*‐test. **** denotes *p* < 0.0001.

All quality statements evaluated demonstrated a significant (*p* < 0.0001) preference for the GPT‐4‐generated reports, based on mean Likert scale ratings from 1 = “*totally disagree*” to 5 = “*totally agree*” when comparing the original surgical reports with the GPT‐4‐generated versions. This included, for example, an enhanced understanding of the surgical indication (2.7 with SD = 0.99 vs. 4.3 with SD = 0.64), the operative steps (2.6 with SD = 0.89 vs. 4.2 with SD = 0.62), and the potential risks and complications (2.6 with SD = 0.89 vs. 4.2 with SD = 0.59) (Figure 3). All patients (*n* = 20) agreed partially or fully that the AI‐generated reports were “a valuable addition to the verbal explanation” of the surgical procedure and outcome, whereas the same was expressed by only 5% (*n* = 1) about the original surgical reports.

### 3.5 Basic linguistic analysis and exemplary excerpt of one representative surgical report

Comparing a representative original surgical report for a laparotomy due to suspected cancer with the GPT‐4‐generated version revealed that the original report was more technical and detailed, including abbreviations and concise, direct sentences. By contrast, the GPT‐4‐generated version was simplified by applying a less technical language and fewer abbreviations. It employed longer sentences with more subordinate clauses (Table 2).

**TABLE 2:** Quantitative linguistic analysis and comparison of one exemplary original surgical report and its simplified version by GPT‐4.

	Original report	GPT‐4 report
Total word count (*n*=)	588	361
Mean word count of sentences (*n*=; SD)	7.41 (5.18)	17.2 (6.8)
Number of technical terms/words (*n*=)	91	3
Number of abbreviations/acronyms (*n*=)	7	0
Number of main clauses (*n*=)	58	22
Number of subordinate clauses (*n*=)	4	17

An exemplary excerpt closely translated from the German original is presented below, describing the abdominal findings after entering the abdominal cavity in both the original and GPT‐4‐generated versions:

#### 3.5.1 Original report

“The following situs is presented: Multiple tumor nodules are visible in the omentum, on the small intestine, and on the peritoneum. A tumor conglomerate involves the left adnexa and a distal part of the ileum, with the mesentery and the lumen of the small intestine also affected. There is a suspicious structure on the gallbladder. The diaphragm, liver, spleen, and Douglas peritoneum are clear. The uterus and right adnexa appear normal. A hardened lymph node is palpable on the left side near the iliac region.”

#### 3.5.2 GPT‐4 report

“Upon entering your abdominal cavity, we observed multiple nodules caused by the tumor disease, located on the peritoneum, small intestine, and omentum—a layer of fat in the abdomen. A larger tumor mass was found near your left adnexal area and the lower part of your small intestine. Your gallbladder showed suspicious changes, but your liver, spleen, and other abdominal areas appeared normal. The lymph nodes in your left pelvic area were enlarged.”

## 4 DISCUSSION

Patients found the GPT‐4‐generated reports significantly easier to understand compared with the original versions and particularly valued the added clarity regarding the indication of the surgery, its outcome, and potential risks. Experts rated the GPT‐4‐generated surgical reports highly for their clarity and comprehensibility but pointed out that these reports were less accurate and complete with regard to clinical and technical details.

The findings of our study align with a growing body of literature that highlights the rationale for using AI or LLM models to enhance patient education and communication. The problem is that the traditional (oral) patient–physician communication can be challenging due to differences in medical knowledge, emotional stress, time constraints, cultural and language barriers, and varying expectations about diagnosis and treatment. Gotlieb et al. conducted a study on patient understanding of medical terminology and discovered significant misunderstandings. For example, patients often misinterpreted common medical terms such as “negative” or “positive” which could result in confusion and miscommunication about their diagnosis or treatment.^15^ Especially in gynecologic oncology, AI chatbots are set to transform how cancer patients will search for information by offering greater accessibility and ease of use, with ChatGPT marking a major paradigm shift in that regard.^10^ Simplified reports generated by AI tools like GPT‐4 may improve patient understanding, making medical information more accessible without the need for specialized preknowledge or academic background.^16^

Several studies examined LLMs like GPT‐4 in different medical settings and reported their ability to simplify complex information for patients. Patel et al. reported that ChatGPT delivers accurate answers to most questions concerning genetic syndromes, genetic testing, and counseling, positioning it as a valuable resource for patients seeking information on genetic counseling in gynecology.^17^ In addition, Braun et al. assessed GPT's responses to questions related to gynecologic oncology and discovered that, while the model excelled in clarity and adherence to guidelines, it consistently showed weaknesses in accuracy and in considering individual patient characteristics.^18^ Our study supports these findings, as clinical experts similarly rated the GPT‐4‐generated surgical reports lower in terms of accuracy and completeness, despite giving them high ratings for understandability.

It is important to note that the method of prompting significantly influences the performance of LLMs. This has led to the emergence of the discipline of “prompt engineering” which focuses on optimizing prompts for medical applications.^13^, ^19^ Comparing different prompting strategies, such as *Input–Output Prompting*, *Zero‐Shot Chain of Thought*, or *Reasoning Optimization Techniques*, revealed significant differences in the quality of responses to medical questions.^12^ Furthermore, the comparison demonstrated that different prompts have varying effects across multiple LLMs. An appropriate prompt, including relevant examples, can enhance the accuracy of responses. Since we utilized in our project the *Input–Output Prompting*, it is conceivable that modified prompting strategies could address the criticism raised by clinical experts regarding the lack of accuracy and completeness in clinical and technical details.

Previous studies have shown trends of improvements in the performance of LLMs across various medical domains. A recent systematic review by Griewing et al. compared various LLMs, including GPT‐4, Llama2, and Google's Bard, with regard to treatment recommendations for complex breast cancer patient profiles. GPT‐4 consistently outperformed its predecessors and competitors.^20^ Lukac et al. found that GPT‐4's treatment recommendations for early‐stage breast cancer aligned closely with expert decisions when clear clinical algorithms were applied. However, the model faced challenges in handling more complex, nuanced cases, where it struggled to provide comprehensive and clinically precise recommendations.^21^ Accordingly, we found that GPT‐4 excelled at conveying general surgical outcomes, but—according to our clinical experts—lacked the necessary depth for detailed and accurate responses. Published literature also supports our view that AI‐driven models, such as GPT‐4, can help bridge communication gaps in healthcare by simplifying medical jargon into more accessible and understandable formats for patients.^22^, ^23^ A notable finding of our study is that 80% of patients indicated that they did not intend to read the original surgical report. This reluctance may be attributable to two main factors. First, the conventional, technical format of surgical documentation often fails to address the information needs of patients; the specialized language and detailed clinical data can be overwhelming and inaccessible to those without a medical background. Second, there is often limited direct and easy access to these reports in routine clinical practice, which further discourages patients from seeking them out. Together, these factors suggest that the current approach to surgical documentation may not be optimal for patient engagement, underscoring the potential value of developing more accessible, patient‐friendly versions of medical reports.

In another comparative analysis by Ocakoglu et al. different LLMs exhibited varying levels of accuracy in responding to questions on patient information for pelvic organ prolapse, and ChatGPT provided the most comprehensive answers.^24^ Our patients overwhelmingly favored the simplified surgical reports generated by GPT‐4 over the original, more technical versions. Based on our basic linguistic analysis and evaluations by clinical experts, it is evident that the simplification of the surgical report occurs in two ways. First, the language is simplified by removing complex syntax and technical terminology. Second, the amount of information and level of detail is reduced. Overall, the simplified version adopts a more “prosaic” writing style, resembling the kind of language patients are accustomed to in their everyday reading. Therefore, the lower *completeness* ratings given by experts to the AI‐generated version may not be a flaw but an inherent result of the simplification process.

However, the issue of accuracy in medical content generated by GPT remains a recurring controversial topic in the literature. While GPT‐4 and similar models excel at text simplification, their tendency to omit essential clinical details or introduce inaccuracies has been widely documented. Clinicians highlighted the problem of “hallucinations” in GPT‐4's responses, particularly in more complex medical scenarios.^18^ This occurs when the AI model generates content that is not grounded in real data or factual information, but instead fabricates details to fill gaps, often presenting these as if they were true and reliable. Such “hallucinations” can result in false statements, invented facts, or completely fictional concepts, even if the language appears convincing and well‐structured.^25^ This issue arises from the fact that GPT lacks true comprehension of the information it processes; instead, it relies on pattern recognition within its training data, without the ability to verify the accuracy of the content. Emerging *reasoning models*, which go beyond simple pattern recognition, may address these shortcomings and lead to improved comprehension and higher‐quality outputs. Furthermore, the most recent medical findings, not yet included in the larger datasets used for LLM training, remain inaccessible to the AI. This is especially critical in the rapidly advancing field of gynecologic oncology treatment. While GPT‐4 has been updated to incorporate information from internet search and provide links and sources, this feature is only partially beneficial, as the AI cannot reliably assess the validity and quality of the information retrieved. Moreover, patients who use LLMs for medical education or information are also most likely unable to evaluate the quality or relevance of the sources. Sarangi et al. tested the ability of GPT‐3.5 to simplify complex radiological reports to improve understanding among healthcare professionals and patients. The results demonstrated that GPT‐3.5 effectively removed technical jargon while preserving essential diagnostic information, achieving high accuracy in summarizing content (up to 94%), though it was less effective in drawing therapeutic consequences.^26^ This aligns with our study, where experts noted gaps in the completeness of the AI‐generated surgical reports, emphasizing the necessity of human oversight when integrating such tools into medical practice.

A key strength of our study is its focus on real‐world clinical settings and cases, allowing patients to review their own simplified surgical reports shortly after their operations. This immediate feedback captures the patients' initial understanding of the procedures. Furthermore, the recruitment of clinical experts in evaluating the GPT‐4‐generated reports provides an essential layer of analysis. However, several limitations deserve consideration. First, the relatively small sample size (*n* = 20) may restrict the generalizability of our findings, as larger studies could offer more robust insights into the effectiveness of AI‐generated reports across more varying patient cohorts (for example, with regard to age or the type and outcome of the operation). The significant improvement in the comprehension of the surgical reports may be influenced by the fact that these reports were not primarily written for educational purposes. Until now, surgical reports have mainly served to document and ensure the traceability of surgical procedures for medical colleagues and to provide adequate legal certainty.

Additionally, our study relied on subjective expert evaluations of the AI‐generated reports. While these assessments are valuable, they may not fully account for all aspects of accuracy and completeness in a quantitative way. Future research could also explore more objective measures of clinical benefits in patient education when using AI, such as directly assessing the patients' knowledge and retention of relevant information through a quiz‐style study format. It is important to note that our study exclusively utilized GPT‐4, as a state‐of‐the‐art LLM at the time of the analysis. Since then, newer models, such as GPT‐4o and o1 by OpenAI, have been introduced and are already being explored for medical applications^27^, ^28^ Furthermore, other platforms, including Claude 3.5 Sonnet, Llama 3, and Gemini, are now available. Studies comparing these platforms continue to demonstrate GPT‐4 as a highly effective LLM.^29^ However, it remains open for discussion whether other models might have performed differently in the context of our specific task. Therefore, our findings are not directly transferable to these newer models.

The findings of this study may have relevant implications for both clinical practice and future research. In the medium term, AI‐generated, simplified, patient‐friendly surgical reports—as well as any other medical documentation—could be integrated into routine and automated postoperative care and standardized (digital) discharge management, allowing patients to better understand their procedures and medical outcomes. One can envision a future clinical setting where a comprehensive, integrated, and patient‐friendly medical report—automatically generated with AI access to all relevant digital medical records from the treatment period (including surgical reports, physical examinations, imaging, blood results, etc.)—becomes a routine part of patient care. This could be particularly beneficial for patients with lower health literacy who may struggle with traditional medical documentation or oral explanations in a stressful and time‐critical clinical setting. Simplified reports may also reduce the burden on healthcare providers, as patients who better understand their treatment may require fewer follow‐up consultations for clarification.

One aspect not explored in this study is the potential to use LLMs interactively as a real *chatbot* that could enable patients to ask personalized follow‐up questions. This could be implemented seamlessly with online access to medical reports, allowing patients to interact with the AI in real time. The patients may ask questions and receive personalized responses, further enhancing their understanding of the medical information. Additionally, modifying the prompts to align better with the individual patient's educational level could improve understanding and make the information more accessible.

However, as our findings and the existing literature indicate, the clinical accuracy of AI‐generated reports remains a concern. To safely integrate these tools into healthcare, further advancements are needed to enhance the depth and precision of the content produced by AI models like GPT‐4. Future research should focus on refining these models to ensure that they provide both simplified and clinically sound information. Examining the long‐term impact of AI‐generated reports on patient outcomes could also be valuable, particularly, to assess whether enhanced understanding contributes to better treatment adherence, reduced anxiety, or greater patient satisfaction.

## 5 CONCLUSION

While AI‐driven simplification of medical reports holds promise for improving patient education, careful attention must be paid to ensuring that accuracy and completeness are not compromised in the pursuit of clarity. As AI technologies continue to evolve, their role in healthcare will likely expand, but their use must be accompanied by rigorous oversight and ongoing research to fully realize their potential in clinical practice.

## AUTHOR CONTRIBUTIONS

**Maximilian Riedel:** Conceptualization, data curation, methodology, formal analysis, project administration, visualization, and writing—original draft. **Bastian Meyer:** Methodology, formal analysis, project administration, and writing—original draft. **Raphael Kfuri Rubens:** Supervision, and writing—original draft. **Caroline Riedel:** Formal analysis and writing—original draft. **Niklas Amann:** Supervision, formal analysis, and writing—original draft. **Marion Kiechle:** Supervision and writing—original draft. **Fabian Riedel:** Conceptualization, project administration, supervision, and writing—original draft.

## CONFLICT OF INTEREST STATEMENT

None.

## ETHICS STATEMENT

An ethics approval was obtained from the ethics committee of the Technical University of Munich (ref: 2024‐499‐S‐CB) on October 30, 2024. Participation in the study was entirely voluntary. Written informed consent had been obtained from all participants before the study.

## AI USAGE

During the preparation of this work, the authors used GPT‐4 for English grammar and spelling correction and general language editing to improve the readability and language of the work. After using this tool, the authors reviewed and edited the content as needed and took full responsibility for the content of the publication.

## Supporting information


**Appendix S1.** Exemplary surgical report (translated automatically from German to English by GPT‐o3 with the prompt to translate as closely to the original as possible).

**Appendix S2.** Questionnaire.

## Data Availability

All (raw) data and material are available upon reasonable request to the corresponding author.
