# Supplementary material for: AI‐driven simplification of surgical reports in gynecologic oncology: A potential tool for patient education
Source: Acta Obstet Gynecol Scand. 2025 May 14;104(7):1373–81. doi: 10.1111/aogs.15123 (PMC12144574; doi:10.1111/aogs.15123)
Supplement: Supplementary file 1 — Appendix S1. Exemplary surgical report (translated automatically from German to English by GPT‐o3 with the prompt to translate as closely to the original as possible). [file AOGS-104-1373-s001.docx]

**Exemplary surgical report (translated automatically from the German to English by GPT-o3 with the prompt to translate as closely to the original as possible)**

Original report

*Diagnosis:*

Serous high-grade endometrial carcinoma

*Operation Type:*Laparotomy: Hysterectomy, bilateral adnexectomy, infragastric omentectomy, pelvic and paraaortic lymphadenectomy

*Course of the Operation:*
The patient was positioned in a flat Steinschnitt position, disinfected and sterilely draped, with disinfection of the vagina and swabbing with a stick swab. A transurethral bladder catheter was placed. A team time-out was performed with the anesthesia team. A median longitudinal laparotomy was made from the symphysis pubis up to 4 cm below the xiphoid process, and the abdomen was opened in layers in the usual manner. The surgical drapes were then applied.

The following situs was observed:

- Right ovary: unremarkable
- Left ovary: approximately 10 cm, mobile, round, livid tumor originating from the ovary
- Uterus: unremarkable

The uterus was grasped with two blunt clamps. The peritoneum lateral to the right adnexa was opened up to the round ligament, followed by dissection and ureterolysis. The ovarian suspensory ligament was identified and undermined, and the adnexa were detached from the ovarian suspensory ligament at the level of the linea terminalis. The same procedure was performed for the left adnexa.

The round ligaments on both sides were identified and detached from the uterus. The bladder peritoneum was incised and the uterus dissected in the area of the plica vesicouterina. The bladder was pushed caudally and dissected down to a point beyond the cervix. Next, the uterine vessels were identified bilaterally and detached. The parametria were detached bilaterally from the uterus—after distancing the bladder and rectum using Zeppelin clamps—and ligated with Vicryl-1 sutures. The ureter was noted to be widely separated.

The uterus was detached from the vagina using Zeppel clamps and ligated with Vicryl-1 sutures. For descent prophylaxis, the vagina was fixed to the sacrouterine ligaments, and the vagina was closed after disinfection.

Subsequently, the peritoneum lateral to the left adnexa was opened up to the round ligament with dissection and ureterolysis. The ovarian suspensory ligament was identified and undermined, and the adnexa were detached from it at the level of the linea terminalis. The same procedure was performed for the right adnexa.

The pelvic lymphadenectomy on the right side was then initiated. The common and external iliac vessels were identified up to the inguinal ligament. The genitofemoral nerve was identified and neurolyzed. The internal iliac artery was identified along the course of the umbilical ligament. Lymph node–fat tissue was excised from the area of the common, external, and internal iliac vessels as well as from the interiliac region.

Next, the obturator fossa and the obturator nerve were identified. Neurolysis of the obturator nerve was performed and lymph node–fat tissue was excised from this area. Meticulous hemostasis was achieved. The pelvic lymphadenectomy on the left was performed analogously.

The colon frame was identified and the greater omentum was detached from the transverse colon with meticulous hemostasis. The greater omentum was then detached on the left towards the spleen. The bursa omentalis was entered, which appeared unremarkable on inspection and palpation; the vessels in the area of the middle colic vessels were carefully preserved and meticulously dissected. The greater omentum was detached from the greater curvature of the stomach below the arcades of the gastroepiploic vessels with meticulous hemostasis. The operative field was bloodless.

The peritoneum in the right paracolic gutter was split and the cecal pole mobilized. The ovarian vessels and the ureter were identified cranially. The peritoneum was opened at the base of the small bowel mesentery with meticulous hemostasis towards the left shoulder. Next, the peritoneum in the left paracolic gutter was split, and the bowel was easily exteriorized in front of the abdominal wall.

The inferior vena cava, the abdominal aorta with the inferior mesenteric artery were identified. The aortic bifurcation was visualized and mobilized up to the origin of the left renal vein. The right ureter was identified cranially. Lymph node–fat tissue was excised beginning at the left renal vein up to the aortic bifurcation, preserving all previously identified structures.

After an abdominal lavage and meticulous hemostasis (with no bleeding observed), the previously placed abdominal towels and surgical drapes were removed. An Easy-Flow drainage was placed in the Douglas pouch and fixed with a suture. The peritoneum and the abdominal wall were closed with a continuous PDS loop suture after meticulous hemostasis. The skin was closed with staples, and a dressing was applied.

The urine was clear and copious. The patient was correctly positioned throughout and experienced an unremarkable anesthesiological course. Following uneventful extubation, the patient was transferred hemodynamically stable to the recovery room, where a repeat anesthesia consultation was performed. According to the OR nurse, all instruments, swabs, and towels were accounted for.

GPT-4 report

You underwent an operation to treat a rare form of uterine cancer that is classified as serious and aggressive. To ensure that the cancer is completely removed, the doctors decided to remove not only the uterus but also the ovaries, the surrounding tissue, and a portion of the abdominal fat known as the net. In addition, lymph nodes in the pelvic and abdominal regions were examined to determine whether the cancer had spread.

At the beginning of the operation, you were positioned to provide the doctors with the best possible access to the surgical areas. Your abdomen was carefully disinfected to minimize infection risks, and a catheter was inserted to empty your bladder during the operation. A safety check was performed with the anesthesia team before a longitudinal incision was made down the middle of your abdomen to expose the internal organs.

The doctors found that one of your ovaries was enlarged by a large tumor. The uterus itself appeared normal. Using special instruments, the uterus was carefully grasped, and the surrounding tissue, including the ovaries, was exposed and removed. Particular care was taken to preserve nearby structures such as the ureters and blood vessels.

The bladder was carefully separated from the lower part of the uterus to gain access to the blood vessels supplying the uterus. These blood vessels were cut and securely sealed to prevent bleeding. The uterus was then separated from the vagina and removed.

To reduce the risk of a renewed descent of the pelvic organs, the upper part of the vagina was attached to the surrounding structures. The net—a portion of fat tissue in the abdomen—was also removed, as it might contain cancer cells.

The removal of the lymph nodes, an important measure for assessing the spread of the cancer, was carried out both in the pelvic region and along the major blood vessels in the abdominal cavity. This helps to more accurately determine the stage of the cancer and to assess the necessity of further treatment.

After all the required parts were removed and the surgical site was thoroughly inspected, the abdominal incision was carefully closed. The operation ended without any unexpected problems, and you were transferred to the recovery room for further monitoring.
