# Supplementary material for: AI‐driven simplification of surgical reports in gynecologic oncology: A potential tool for patient education
Source: Acta Obstet Gynecol Scand. 2025 May 14;104(7):1373–81. doi: 10.1111/aogs.15123 (PMC12144574; doi:10.1111/aogs.15123)

**´**

Klinikum rechts der Isar

Technische Universität München

Questionnaire 1:

Introduction

**8. Do you currently have any outstanding**

**questions regarding the procedure or out-**

**come of the surgery?**

General Information

**1. Age: ____ years**

[ ] Yes

[ ] No

**2. Gender:**

[ ] Female

[ ] Male

**9. In addition to the information provided,**

**I also informed myself about the proce-**

**dure through other sources (literature, in-**

**ternet, acquaintances, etc.).**

[ ] A lot

[ ] A little

[ ] Not at all

[ ] Other

**3. Highest Level of Education:**

[ ] Primary School

[ ] Secondary School (lower level)

[ ] Secondary School (intermediate level)

[ ] High School Diploma

[ ] University Degree

Technology and Internet Use

[ ] Other

**10. How often do you use the Internet per**

**week?**

[ ] Daily

Questions about the Surgery

**4. Date of Surgery:** _________

[ ] Several times per week

[ ] Once a week

[ ] Less frequently

**5. How well did you feel informed about**

**the procedure before the surgery (on a**

**school grading scale)?**

[ ] Very well (1)

[ ] Well (2)

[ ] Satisfactory (3)

**11. Which devices do you regularly use to**

**access the Internet? (Multiple selection**

**possible)**

[ ] Smartphone

[ ] Laptop

[ ] Sufficient (4)

[ ] Insufficient (5)

[ ] Tablet

[ ] Desktop Computer

[ ] None of the above

**6. Were you informed about the proce-**

**dure and outcome of the surgery?**

[ ] Yes

[ ] No

[ ] Don’t know

**12. Which internet applications do you**

**regularly use? (Multiple selection possi-**

**ble)**

**7. How well do you feel informed about**

**the procedure and outcome of the surgery**

**(on a school grading scale)?**

[ ] Very well (1)

[ ] Well (2)

[ ] Satisfactory (3)

[ ] Email

[ ] Social Media (e.g., Facebook, Instagram)

[ ] Online Messaging Services (e.g.,

WhatsApp, Telegram)

[ ] Online Shopping

[ ] Searching for Health Information

[ ] Other (please specify: __________)

[ ] poorly (4)

[ ] very poorly (5)

Seite 1 von 6


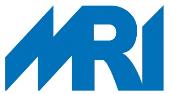

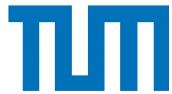

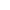


Klinikum rechts der Isar

Technische Universität München

**13. Have you had prior experience using**

**chat-based tools or AI technologies to sim-**

**plify medical information?**

[ ] Yes

[ ] No

**14. Are you familiar with the ChatGPT ap-**

**plication?**

[ ] Yes

[ ] No

**15. Agreement/Disagreement with the**

**statement: "Artificial intelligence will play**

**a greater role in the future of medicine**

**and patient care."**

[ ] Strongly agree

[ ] Partially agree

[ ] Undecided

[ ] Partially disagree

[ ] Strongly disagree

Seite 2 von 6


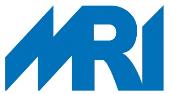

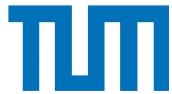


Surgery Report 1:

Klinikum rechts der Isar

Technische Universität München

Please read Surgery Report 1 and then answer the questions in "Questionnaire 2."

Seite 3 von 6


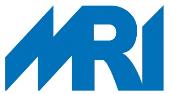

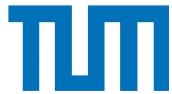


Questionnaire 2:

Surgery Report 1

Klinikum rechts der Isar

Technische Universität München

**20. Agreement/Disagreement with the**

**statement: "After reading the surgery re-**

**port, I have a better overall understanding**

**of my surgery."**

**16. Agreement/Disagreement with the**

**statement: "The surgery report helped me**

**better understand the reasons for my sur-**

**gery."**

[ ] Strongly agree

[ ] Strongly agree

[ ] Partially agree

[ ] Partially agree

[ ] Neutral

[ ] Neutral

[ ] Partially disagree

[ ] Partially disagree

[ ] Strongly disagree

[ ] Strongly disagree

**21. Agreement/Disagreement with the**

**statement: "The surgery report is a valua-**

**ble addition to the verbal explanation."**

[ ] Strongly agree

[ ] Partially agree

[ ] Neutral

**17. Agreement/Disagreement with the**

**statement: "The surgery report helped me**

**better understand the individual surgical**

**steps and procedures."**

[ ] Strongly agree

[ ] Partially agree

[ ] Partially disagree

[ ] Neutral

[ ] Strongly disagree

[ ] Partially disagree

[ ] Strongly disagree

**22. Estimate the percentage of the surgery**

**report you understood overall (please**

**provide a whole number; 0-100%)**

_______ %

**18. Agreement/Disagreement with the**

**statement: "The technical terms used in**

**the surgery report made understanding**

**more difficult."**

[ ] Strongly agree

[ ] Partially agree

**23. Agreement/Disagreement with the**

**statement: "The surgery report helped me**

**better understand the risks and possible**

**complications of the procedure."**

[ ] Strongly agree

[ ] Neutral

[ ] Partially disagree

[ ] Strongly disagree

[ ] Partially agree

[ ] Neutral

[ ] Partially disagree

[ ] Strongly disagree

**19. Agreement/Disagreement with the**

**statement: "The surgical procedure de-**

**scribed in the surgery report matches my**

**understanding from the pre-surgery con-**

**sultation."**

[ ] Strongly agree

[ ] Partially agree

[ ] Neutral

[ ] Partially disagree

[ ] Strongly disagree

Seite 4 von 6


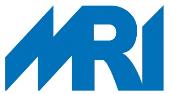

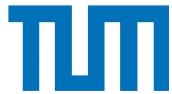


Surgery Report 2:

Klinikum rechts der Isar

Technische Universität München

Please read Surgery Report 2 and then answer the questions in "Questionnaire 3".

Seite 5 von 6


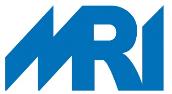

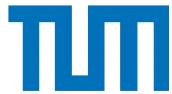


Klinikum rechts der Isar

Technische Universität München

Questionnaire 3:

Surgery Report 2

**20. Agreement/Disagreement with the**

**statement: "After reading the surgery**

**report, I have a better overall under-**

**standing of my surgery."**

[ ] Strongly agree

[ ] Partially agree

[ ] Neutral

**16. Agreement/Disagreement with the**

**statement: "The surgery report helped**

**me better understand the reasons for**

**my surgery."**

[ ] Strongly agree

[ ] Partially agree

[ ] Neutral

[ ] Partially disagree

[ ] Strongly disagree

[ ] Partially disagree

[ ] Strongly disagree

**21. Agreement/Disagreement with the**

**statement: "The surgery report is a val-**

**uable addition to the verbal explana-**

**tion."**

[ ] Strongly agree

[ ] Partially agree

[ ] Neutral

[ ] Partially disagree

[ ] Strongly disagree

**17. Agreement/Disagreement with the**

**statement: "The surgery report helped**

**me better understand the individual**

**surgical steps and procedures."**

[ ] Strongly agree

[ ] Partially agree

[ ] Neutral

[ ] Partially disagree

[ ] Strongly disagree

**22. Estimate the percentage of the sur-**

**gery report you understood overall**

**(please provide a whole number; 0-**

**100%)**

**18. Agreement/Disagreement with the**

**statement: "The technical terms used in**

**the surgery report made understanding**

**more difficult."**

_______ %

[ ] Strongly agree

[ ] Partially agree

[ ] Neutral

[ ] Partially disagree

[ ] Strongly disagree

**23. Agreement/Disagreement with the**

**statement: "The surgery report helped**

**me better understand the risks and pos-**

**sible complications of the procedure."**

[ ] Strongly agree

**19. Agreement/Disagreement with the**

**statement: "The surgical procedure de-**

**scribed in the surgery report matches**

**my understanding from the pre-surgery**

**consultation."**

[ ] Partially agree

[ ] Neutral

[ ] Partially disagree

[ ] Strongly disagree

[ ] Strongly agree

[ ] Partially agree

[ ] Neutral

[ ] Partially disagree

[ ] Strongly disagree


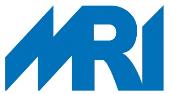

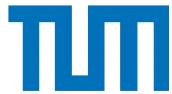

Supplement: Supplementary file 2 — Appendix S2. Questionnaire. [file AOGS-104-1373-s002.docx]
